# Sex-Dependent Effects of Aging and Insulin Resistance on Skeletal Muscle Function and Structure in Rats

**DOI:** 10.3390/ijms26146783

**Published:** 2025-07-15

**Authors:** Patricia Sosa, Javier Angulo, Alberto Sánchez-Ferrer, Maria Carmen Gómez-Cabrera, Argentina Fernández, Leocadio Rodríguez-Mañas, Mariam El Assar

**Affiliations:** 1 Fundación para la Investigación Biomédica del Hospital de Getafe, 28905 Getafe, Spainasferrer@salud.madrid.org (A.S.-F.); 2 Centro de Investigación Biomédica en Red de Fragilidad y Envejecimiento Saludable (CIBERFES), Instituto de Salud Carlos III, 28029 Madrid, Spain; javier.angulo@hrc.es (J.A.); carmen.gomez@uv.es (M.C.G.-C.); 3 Instituto de Investigación IdiPaz, 28029 Madrid, Spain; 4 Servicio de Histología-Investigación, Unidad de Investigación Traslacional en Cardiología–IRYCIS/UFV, Hospital Universitario Ramón y Cajal, 28034 Madrid, Spain; argentina.fernandez@hrc.es; 5 Freshage Research Group, Department of Physiology, Faculty of Medicine, University of Valencia, Fundación Investigación Hospital Clínico Universitario/INCLIVA, 46010 Valencia, Spain; 6 Servicio de Geriatría, Hospital Universitario de Getafe, 28905 Getafe, Spain

**Keywords:** aging, insulin resistance, sex differences, muscle function, contractile responses, glycolytic fiber, fibrosis

## Abstract

Skeletal muscle function is determinant for maintaining functional performance and independence in older adults. Muscle is a primary target of aging and insulin resistance (IR)—two conditions associated with functional decline. Sex-related differences may influence these effects at structural and functional levels. We aimed to evaluate the individual and combined effects of aging and IR on the function and structure of extensor digitorum longus (EDL) and soleus muscles in male and female rats. Animals aged 3 and 20 months were studied, with IR induced by 8 weeks of 20% fructose in drinking water. Muscle contractility was assessed alongside histological and hormonal analyses. In males, aging impaired EDL and soleus contractile force, free testosterone levels, and muscle mass. IR decreased muscle function only in young animals. In females, aging led to muscle loss without affecting contractile strength, but the combination of aging and IR reduced muscle contraction, decreased estradiol and exacerbated muscle loss. Both sexes showed aging-related loss of EDL glycolytic fibers, altered regenerative capacity, and increased fibrosis. IR alone reduced glycolytic fibers in young animals of both sexes but increased fibrosis only in males. These results highlight sex-specific effects of aging and IR on muscle function, relevant for targeted strategies to prevent and treat age- and IR-related muscle function decline.

## 1. Introduction

Current society is facing a demographic shift marked by increasingly aging populations [1]. However, the increase in life expectancy has not been accompanied by a proportional rise in the number of years lived in good health, which constitutes a significant challenge for health and social systems [2,3].

The aging process is characterized by various highly prevalent changes, including an increase in morbidity and a decline in functional status, which are two interrelated yet distinct conditions. In this context, functional performance in older adults is the factor most strongly linked to quality of life, as well as to the risk of hospitalization, long-term institutionalization, use of social and healthcare resources, and death [4]. The World Health Organization has acknowledged the key role of function in healthy aging and the importance of identifying the factors involved in its preservation or deterioration [5,6]. Consequently, understanding the intrinsic mechanisms underlying age-related functional decline is crucial for establishing preventive, early detection strategies, as well as for identifying new therapeutic targets aimed at improving quality of life in old age and delaying the onset of functional decline.

Skeletal muscle is one of the main systems affected by aging, with muscle function being key to the functional status of older adults. Musculoskeletal alterations, specifically age-related sarcopenia, a condition marked by both structural (atrophy) and functional (weakness) muscle loss, are strongly associated with a significant decline in quality of life, as well as increased morbidity, frailty, and premature mortality [7,8]. Sarcopenic muscle is characterized by a reduction in both the number and size of muscle fibers, particularly affecting type II (glycolytic) fibers, which are primarily responsible for high-intensity and strength-related activities [7,9]. At the sarcomere levels, the loss of contractile proteins is accompanied by their replacement with adipose and fibrous tissue. This structural remodeling, combined with a decline in motor neuron number and capillary density, leads to a significant decrease in muscle contractile capacity [10,11,12].

In recent years, various lines of evidence have pointed to a close relationship between IR and the development of functional decline. In line with this, a negative correlation between IR and gait speed in non-diabetic men aged 50 and over has been revealed [13]. Similarly, quadriceps muscle strength in older non-diabetic adults has been shown to negatively correlate with IR regardless of age, sex, daily physical activity, or glucose intake [14]. Moreover, we have recently reported that IR, determined by the HOMA-IR index, is associated with an increased risk of functional decline in non-diabetic older adults [15]. In animal models, the inhibition of the insulin signaling pathway has been associated with both increased longevity and reduced physical capacity [16,17]. This is, in part, due to the detrimental effects of IR on skeletal muscle. Importantly, skeletal muscle serves as the primary site for insulin-stimulated glucose metabolism, and muscle loss has been associated with IR. In line with this, IR has been consistently related to skeletal muscle loss in both human and animal studies [18,19,20]. Various studies in animal models have shown that IR induced by sucrose-rich diets results in a reduction of lean body mass, increased fat accumulation, and decreased protein synthesis in muscle [18,21,22]. Despite the current evidence, the mechanisms underlying muscle dysfunction associated with insulin resistance in older adults remain unclear.

With aging, both men and women experience a progressive loss of muscle mass and strength; however, the pattern and magnitude of these changes differ between sexes [21,23]. Men generally have greater muscle mass and strength in early life stages but undergo a more accelerated decline with age, increasing their risk of developing sarcopenia. In contrast, women tend to experience a more gradual loss over time [24].

Aging also leads to hormonal and metabolic changes that directly impact muscle health. The decline in sex hormones, testosterone in men and estrogens in women, could impair the maintenance of muscle mass, contributing to the development of sarcopenia [23]. Furthermore, emerging evidence suggests that the impact of IR on muscle physiology is not uniform across all individuals and may vary significantly based on intrinsic factors, such as age and sex. In line with this, a recent study has shown that the ability of IR indexes to indicate the risk of functional deterioration is gender specific [25]. Therefore, understanding these age- and sex-related differences in how IR affects muscle function is crucial for developing more effective therapeutic strategies to improve muscle performance in the aging population.

Hence, the aim of this study was to investigate the individual and combined effects of aging and IR on functional capacity and skeletal muscle composition in two distinct muscle types, the EDL and the soleus, in a rat model and to examine how these effects differ between sexes.

## 2. Results

### 2.1. The Impact of Aging and Insulin Resistance on Hormonal Levels and Muscle Mass in Male and Female Rats

To assess the impact of IR on muscle function in both young and aged animals, 20% D-fructose was administered in drinking water for 8 weeks. Our group previously validated this IR model in young rats [26,27]. The successful induction of IR in young animals was confirmed by a significant increase in the HOMA-IR index compared to age-matched rats (29 ± 7%, *p* = 0.009 and 34 ± 8%, *p* = 0.001 for male and female rats, respectively). In addition, D-fructose also provoked IR in old animals compared to their counterparts (50 ± 10%, *p* = 0.009 and 76 ± 27%, *p* = 0.02; for males and female rats, respectively).

Once IR was established in young and old animals, we proceeded to evaluate the physiological consequences of both aging and IR. Body weight was assessed in all experimental groups. In male rats, a significant increase was observed in both 20-month-old groups (20 M) and 20 M insulin-resistant (20 M IR) compared to young 3-month-old controls (3 M) and 3 M IR. Similarly, aged female rats showed a significant weight gain relative to their younger counterparts. IR-aged females displayed a tendency toward increased body weight compared to their age-matched controls, albeit these differences did not reach statistical significance (Table 1).

The impact of aging on circulating levels of key hormonal biomarkers, such as free testosterone and estradiol, was evaluated using ELISA on serum samples from all the experimental groups. In male rats, although serum-free testosterone levels declined with age, they were not significantly affected by IR. In contrast, a significant decrease in serum estradiol levels was only detected in 20 M IR female rats compared to 3 M females. However, aging did not induce a significant decline in circulating levels of estradiol in female rats without IR (Table 1).

To specifically assess the impact of aging and IR on skeletal muscle mass, the ratio of EDL and soleus muscle mass to total body weight was analyzed across experimental groups. This analysis allowed us to determine potential alterations in muscle composition associated with aging and IR. In male rats, a significant reduction in the percentage of muscle mass of both EDL and soleus muscle was observed in 20 M animals, regardless of IR status, compared to young controls. Notably, IR did not exacerbate muscle mass loss in aged males and had no effect in young 3 M animals. In female rats, the percentage of EDL and soleus muscle mass was also significantly lower in aged animals compared to young ones in both IR and control animals. However, aged IR females showed an additional reduction in EDL and soleus muscle mass compared to their age-matched controls (Table 1). Thus, aging caused a decline in testosterone levels and muscle mass that was not significantly modified by the presence of IR in male rats. In contrast, female rats displayed reduced estradiol levels only when IR was concomitant to aging, a situation that significantly aggravated the muscle mass loss associated with aging itself.

### 2.2. Sex Influences the Impact of Aging and Insulin Resistance on Muscle Function

**Figure 1:**
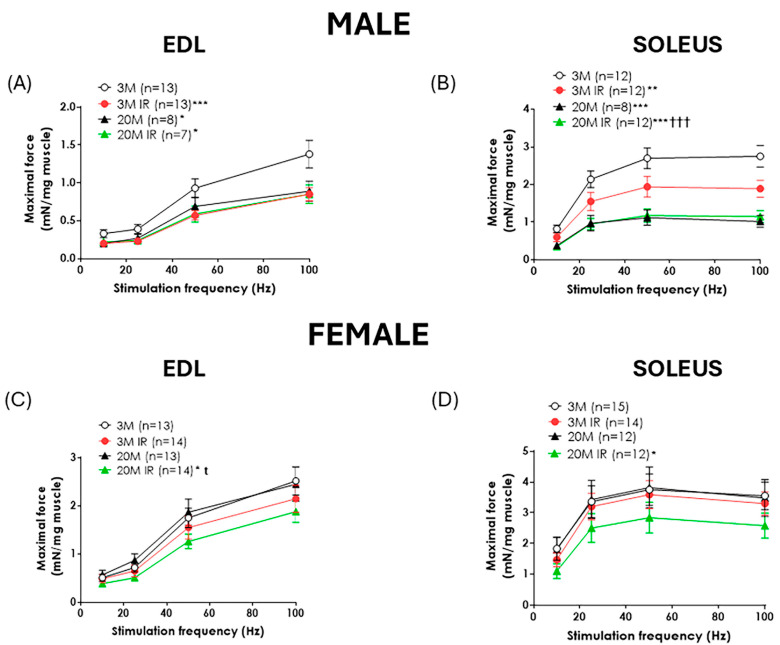
Muscle contraction is affected by age and insulin resistance in a sex-dependent manner. Force–frequency curves (10–100 Hz) of extensor digitorum longus (EDL) and soleus muscles from male (**A**,**B**) and female (**C**,**D**) rats at 3 months (3 M) and 20 months (20 M) of age, in control and insulin-resistant (IR) conditions. Data are expressed as mean ± standard error of the force generated in response to each electrical stimulus normalized by muscle weight. n indicates the number of animals per group. * *p* < 0.05; ** *p* < 0.01, *** *p* < 0.001 vs. 3 M. t < 0.05 vs. 20 M; ††† *p* < 0.001 vs. 3 M IR by a two-factor ANOVA test followed by Bonferroni correction.

Aging caused a significant decrease in contraction force in response to electrical stimulation in both EDL and soleus muscles from male rats (Figure 1A,B). In contrast, no significant age-related differences in contraction force were detected in female rats’ EDL or soleus muscles in response to electrical stimulation (Figure 1C,D). The impact of IR on muscle function was evaluated in all experimental groups. IR resulted in a significant reduction in contraction force in both EDL and soleus muscles in young male rats. Notably, the EDL muscle appeared more sensitive to the effects of IR than the soleus. Indeed, the decline in contractile force of EDL related to IR induction in young animals was comparable to that observed with aging alone. In contrast, the force reduction caused by IR in the soleus from young animals did not reach the impairment produced by aging in this muscle. Unlike what was observed in young male rats, IR did not exacerbate the age-related reduction in muscle contraction in both muscles (Figure 1A,B).

On the other hand, in female rats, the administration of 20% of D-fructose for 8 weeks to young animals did not significantly affect muscle contractility in either the EDL or soleus muscles when compared to young controls (Figure 1C,D). In contrast, the presence of IR in aged rats caused a significant reduction in contraction force in both muscles, even though aging itself did not induce any decline in muscle function (Figure 1C,D). Interestingly, the impact of IR on muscle function was more pronounced in the EDL muscle, as evidenced by a reduction in contraction force in 20 M IR versus 20 M control females (Figure 1C), supporting the idea that the EDL muscle is more sensitive to the effects of IR.

**Figure 2:**
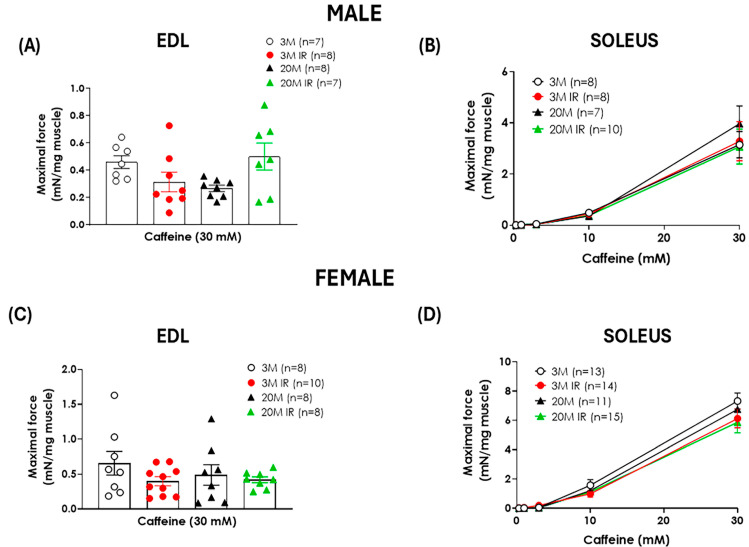
Caffeine response in EDL and soleus muscles is unaffected by aging and insulin resistance in male and female rats. Maximal force responses to 30 mM caffeine stimulation were recorded in EDL muscles (**A**,**C**), and contractile responses to cumulative caffeine contraction (0.3–30 mM) were assessed in soleus muscles (**B**,**D**) from male (**A**,**B**) and female (**C**,**D**) rats at 3 months (3 M) and 20 months (20 M) of age in control and insulin-resistant (IR) conditions. Data are expressed as mean ± standard error of contractile force normalized by muscle weight. n indicates the number of animals per group.

In addition to evaluating muscle function in rats by electrical stimulation, muscle contraction in response to increasing concentrations of caffeine was examined. No significant age-related changes in contraction force in response to caffeine were found in either the soleus or EDL muscles from female or male rats. Likewise, IR did not affect caffeine-induced muscle contraction (Figure 2).

### 2.3. Impact of Aging and IR on Glycolytic Fiber Content in EDL Muscle

**Figure 3:**
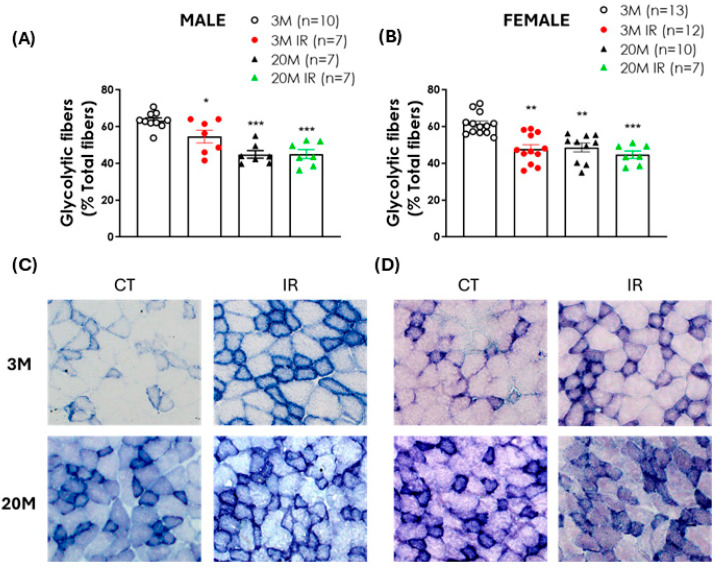
Aging and insulin resistance impact EDL glycolytic fiber content in male and female rats. Percentage of glycolytic fibers in the extensor digitorum longus (EDL) muscle of 3- and 20-month-old control (3 M, 20 M) and insulin-resistant (IR) (3 M IR, 20 M IR) male (**A**) and female (**B**) rats. Representative succinate dehydrogenase (SDH)-stained sections of the EDL muscle of males (**C**) and females (**D**) obtained with 100× magnification: upper left, 3 M; lower left, 20 M; upper right, 3 M IR; lower right, 20 M IR. Oxidative fibers stained purple, and glycolytic fibers appeared lighter. SDH activity staining is expressed as the mean ± standard error of the percentage of glycolytic fibers relative to total fibers. n indicates the number of animals per group. * *p* < 0.05, ** *p* < 0.01, *** *p* < 0.001 vs. 3 M by one-factor ANOVA followed by Tukey’s multiple comparisons test.

To investigate the effect of aging and IR on muscle fiber type content, succinate dehydrogenase (SDH) staining was used. Analysis of the EDL muscles revealed an age-associated shift in fiber composition. Specifically, a significant reduction in the percentage of glycolytic fibers was observed in aged rats compared to young controls, independent of sex or IR status (Figure 3A,B). Moreover, young IR rats showed a marked decrease in the proportion of glycolytic fibers compared to age-matched controls, suggesting that IR may also influence muscle fiber type distribution at an earlier stage of life (Figure 3A,B).

### 2.4. Aging Is a Determinant Factor in Altering Muscle Regenerative Capacity

Hematoxylin/eosin staining was used to assess the number of centrally located nuclei per fiber in both EDL and soleus across experimental groups.

**Figure 4:**
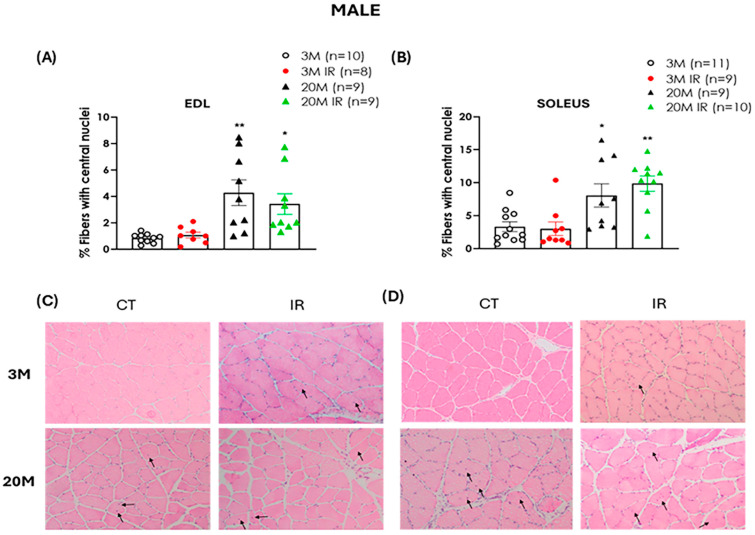
Aging is associated with an increase in the proportion of muscle fibers with central nuclei in male rats. Upper Panel (**A**) displays the percentage (%) of fibers with central nuclei in the EDL muscle of 3- and 20-month-old control (3 M and 20 M) and insulin-resistant (3 M IR and 20 M IR) male rats. The percentage of fibers with central nuclei in the soleus muscle of the same groups is illustrated in panel (**B**). Data are expressed as mean ± standard error of the percentage of fibers with central nuclei relative to the total number of fibers. n indicates the number of animals analyzed. Representative hematoxylin and eosin-stained sections of EDL (**C**) and soleus (**D**) muscles from male rats obtained with 100× magnification: upper left, 3 M; lower left, 20 M; upper right, 3 M IR; lower right, 20 M IR. Arrows indicate fibers with centralized nuclei. * *p* < 0.05, ** *p* < 0.01 vs. 3 M by one-factor ANOVA followed by Tukey’s multiple comparisons test.

A significantly higher percentage of muscle fibers containing centrally located nuclei was observed in aged male rats compared to young male controls in both muscle types analyzed (EDL and soleus) (Figure 4A,B). This increase was evident regardless of the IR status, suggesting that aging itself may contribute to an altered regenerative response. Furthermore, the presence of IR in young animals did not result in an increased number of centrally located nuclei compared to age-matched controls (Figure 4A,B).

**Figure 5:**
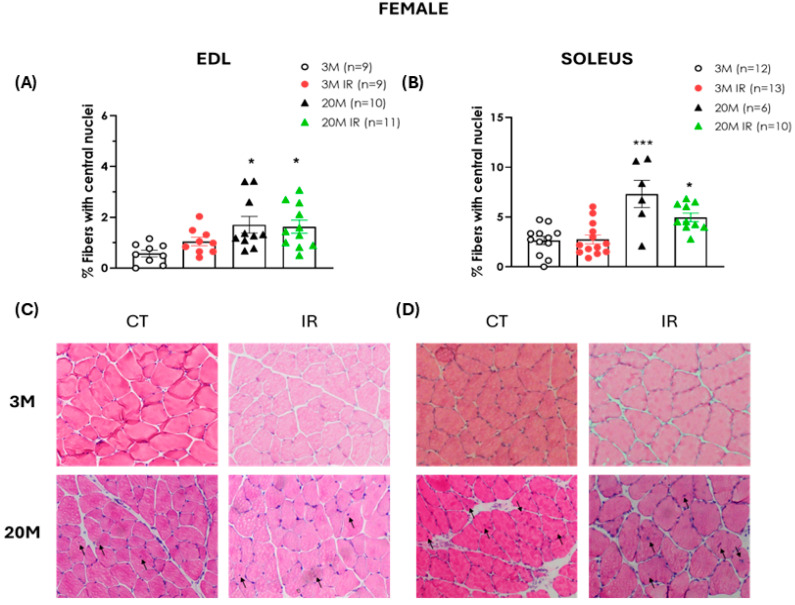
Increased central nuclei number in muscle fibers of aged female rats. Upper Panel (**A**) displays the percentage (%) of fibers with central nuclei in the EDL muscle of 3- and 20-month-old control (3 M and 20 M) and insulin-resistant (3 M IR and 20 M IR) female rats. The percentage of fibers with central nuclei in the soleus muscle of the same groups is illustrated in panel (**B**). Data are expressed as mean ± standard error of the percentage of fibers with central nuclei relative to the total number of fibers. n indicates the number of animals analyzed. Representative hematoxylin and eosin-stained sections of EDL (**C**) and soleus (**D**) muscles from female rats obtained with 100× magnification: upper left, 3 M; lower left, 20 M; upper right, 3 M IR; lower right, 20 M IR. Arrows indicate fibers with centralized nuclei. * *p* < 0.05, *** *p* < 0.001 vs. 3 M by one-factor ANOVA followed by Tukey’s multiple comparisons test.

Similarly, in both EDL and soleus muscles from female rats, aging was associated with a significantly higher percentage of fibers containing centrally located nuclei compared to 3 M controls independently of IR status (Figure 5A,B).

### 2.5. The Impact of Aging and Insulin Resistance on Muscle Fibrosis in Male and Female Rats

**Figure 6:**
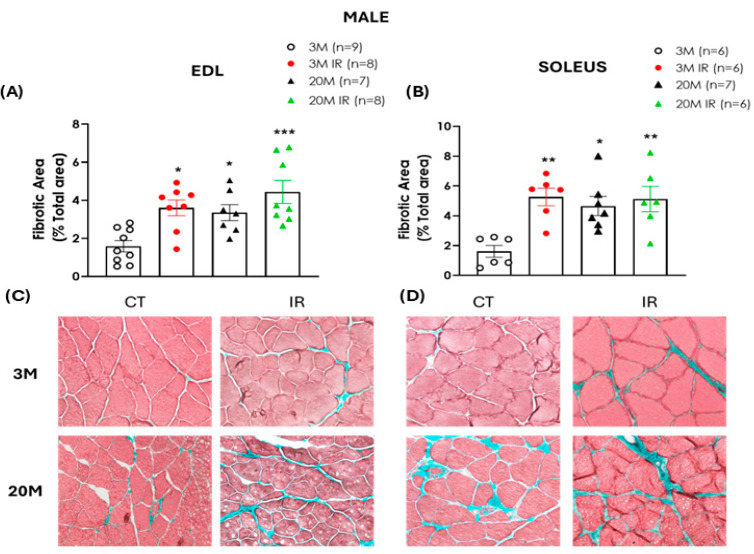
Increased fibrotic area in EDL and soleus muscles of male rats is associated with aging and insulin resistance. Percentage of fibrotic area in the EDL (**A**) and soleus (**B**) muscles of 3- and 20-month-old (3 M and 20 M) male control and insulin-resistant (IR) rats. Representative images of EDL (**C**) and soleus (**D**) muscle sections stained with Masson’s trichrome and obtained with 200× magnification: upper left, 3 M; lower left, 20 M; upper right, 3 M IR; lower right, 20 M IR. In Masson’s staining, collagen fibers are stained green, while muscle fibers are stained red. Fibrosis was expressed as mean ± standard error of the percentage of fibrotic area (green) relative to the total muscle area. n indicates the number of animals per group. * *p* < 0.05, ** *p* < 0.01, *** *p* < 0.001 vs. 3 M by one-factor ANOVA followed by Tukey’s multiple comparisons test.

To assess the potential accumulation of connective tissue in skeletal muscles, Masson’s trichrome staining was performed to visualize and quantify fibrosis through the detection of collagen fibers. In both EDL and soleus muscles of male rats, aging was associated with a significant increase in fibrotic area compared to the young control group, independently of their IR status. In contrast, the presence of IR in young animals was related to a significant elevation in the percentage of fibrosis when compared to their age-matched controls (Figure 6A,B).

**Figure 7:**
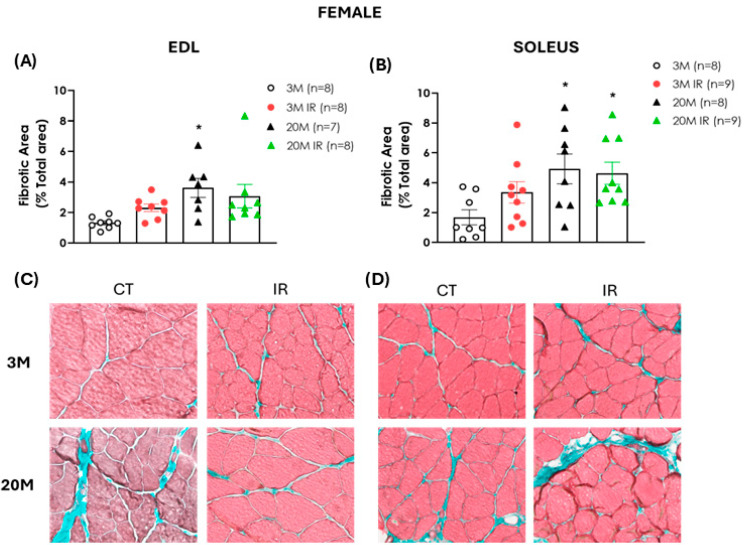
Increased fibrotic area in the EDL and soleus muscles of female rats is associated solely with aging. Percentage of fibrotic area in the EDL (**A**) and soleus (**B**) muscle of 3- and 20-month-old (3 M, 20 M) control and insulin-resistant (IR) rats. Representative images of EDL (**C**) and soleus (**D**) muscles stained with Masson’s trichrome and obtained with 200× magnification: upper left, 3 M female; lower left, 20 M female; upper right, 3 M IR female; lower right, 20 M IR female. Collagen fibers are stained green, and muscle fibers are red. Fibrosis was expressed as mean ± standard error of the percentage of fibrotic area (green) relative to the total muscle area. n indicates the number of animals evaluated. * *p* < 0.05 vs. 3 M by one-factor ANOVA followed by Tukey’s multiple comparisons test.

Similar to the findings in male rats, female animals also exhibited an age-related increase in muscle fibrosis. Specifically, a significant expansion of the fibrotic area was observed in both the EDL and soleus muscles of 20-month-old control females compared to the 3-month-old control rats. Additionally, this increase was also evident in the soleus of the 20 MIR rats. In contrast to the findings in male animals, no significant differences were observed between the two young female groups (Figure 7A,B), suggesting that muscles from female rats are less susceptible to developing fibrosis in response to IR.

## 3. Discussion

In the present study, we demonstrate that aging and IR affect skeletal muscle functional capacity in a sex-dependent manner. In male rats, aging significantly impaired contractile force in the EDL and soleus muscles, accompanied by decreased free testosterone levels and reduced muscle mass. While IR reduced muscle contraction capacity in young animals, it did not exacerbate the age-related decline in muscle function. In females, aging led to a loss of muscle mass without affecting contractile strength, suggesting preserved function. However, when IR was concomitant to aging, a significant impairment of muscle contraction was observed, which was associated with a decline in estradiol levels and exacerbated muscle mass loss. Additionally, structural and compositional changes, including reduced EDL glycolytic fiber content, altered regenerative response, and increased muscle fibrosis, were found with aging in both sexes. Notably, IR in young animals led to a reduced proportion of glycolytic fibers in the EDL muscle in both sexes, while significantly increasing muscle fibrosis exclusively in male rats.

Skeletal muscle is one of the main systems affected by the aging process. Aging is linked to a gradual decline in skeletal muscle mass, even in the absence of disease [7,28]. Skeletal muscle structure and function are highly sex-specific, with approximately 3000 genes differentially expressed between males and females [29]; therefore, extrapolating findings from males to females without accounting for the sex-specific context of skeletal muscle aging is inadequate. Our findings reinforce the notion that aging is associated with a significant loss of muscle mass in both sexes, affecting the EDL muscle, which mainly contains fast-twitch fibers, as well as the soleus muscle, predominantly containing slow-twitch fibers [30].

The loss of muscle mass is often accompanied by a decline in muscle function in older adults [20,21,23]. This deterioration in muscle health contributes directly to reduced physical performance, compromising independence and quality of life in older adults [31]. In this sense, our results reveal important sex-dependent differences in the impact of aging on muscle performance. While aging caused a significant decrease in contraction force in response to electrical stimulation in both EDL and soleus muscles from male rats, aging itself did not result in impairment of contractile responses in muscles from female rats. These results are consistent with previous studies showing that muscle mass loss and function in males are primarily driven by aging [21]. In contrast to our results, previous studies evaluating the impact of aging in females have reported an aging-related decrement in the maximal force-generating capacity of soleus muscles [32]. However, there is evidence demonstrating that age-related decline in muscle power and force is steeper in men than in women [33], while a greater loss of muscle function in aged male mice with respect to female counterparts has also been observed [34]. Moreover, an aging-induced increase in muscle fatigability was detected in male but not in female rats [35,36]. The present results would support the greater susceptibility to muscle function decline with aging in males.

Growing evidence suggests a strong link between IR and functional decline, a key factor in healthy aging. This relationship is largely attributed to the detrimental effects of IR on skeletal muscle. Numerous human and animal studies have consistently associated IR with muscle loss [18,20]. In animal models, IR induced by sucrose-rich diets has been shown to reduce lean body mass, increase fat accumulation, and decrease protein synthesis in muscle [18,21,22]. Sex also influences the impact of IR on muscle function [37]. Women generally maintain higher insulin sensitivity than men throughout life [38], and recent evidence has shown that the predictive value of IR for functional decline is sex-specific [25].

In this context, we analyzed the impact of IR alone or concomitant with aging on skeletal muscle function in female and male rats using a well-characterized non-obese insulin resistance model in fed-fructose rats [26]. The administration of 20% fructose in drinking water for eight weeks provoked IR not only in young animals, as we previously reported [26,27], but also in aged animals, as evidenced by higher HOMA scores when compared to age-matched controls, a fact observed in both sexes. However, the impact of IR on muscle mass and contractile force in response to electrical stimulation differed between sexes. IR resulted in reduced muscle contractile force in both the EDL and soleus muscles from young male rats, indicating that metabolic dysfunction can negatively impact muscle function even in the absence of aging. In contrast, aging-related muscle impairment in male rats was not worsened by the presence of IR. These findings suggest that, while aging is the main determinant of muscle deterioration later in life, IR can independently initiate or exacerbate this decline at earlier stages [39]. By contrast, IR did not exacerbate muscle contractile deficits in aging animals, implying that aging may overshadow the additional impact of metabolic dysfunction at this stage. Notably, the EDL muscle appeared more susceptible to IR than the soleus. In young animals, the reduction in EDL contractile force caused by IR was comparable to that observed with aging alone, whereas the effect of IR on the soleus did not reach the level of impairment seen with aging. This concept aligns with previous evidence suggesting that glycolytic muscles, such as the EDL, are more vulnerable to IR-induced imbalances in energy supply and oxidation than oxidative muscles such as the soleus [40]. Furthermore, certain proteins have been reported to be differently expressed in these muscles, with some proteins regulated in opposite ways [41].

Nevertheless, the influence of IR on muscle function in female rats presented a quite different pattern. While the presence of IR in young females did not impact muscle function, a significant decline in the contractile capacity of both the EDL and soleus was observed only in the aged IR group, indicating that the functional impairment of skeletal muscle in females emerges when aging and IR are present. Furthermore, although IR did not cause muscle mass loss in young male and female animals, a further decrease in muscle mass beyond the effect of aging itself was produced by the presence of IR in old female rats.

A significant difference between males and females that likely contributes to the distinct responses observed in muscle pathologies is the varying concentration of circulating hormones. Although these are not the unique determinants of sex differences in muscle health, their interactions appear to play a relevant role in muscle quality and condition [42].

In our study, aged male rats that exhibited reduced muscle mass and contractile capacity in both fast-twitch EDL and slow-twitch soleus muscle also presented lower testosterone levels compared to young animals. Previous studies have reported an impact of testosterone on muscle mass and metabolic health. With aging, decreasing testosterone levels play a key role in the reduction of muscle mass and strength [23]. This decline occurred independently of IR, suggesting that muscle mass deterioration in males is primarily driven by aging.

Notably, female rats displayed muscle function impairment only when IR was concomitant to aging, a situation that significantly aggravated the muscle mass loss related to aging itself. Interestingly, it is only in these aged and insulin-resistant female rats that the levels of estradiol significantly decreased, suggesting that hormone decline associated with IR may be a decisive factor in functional impairment in aged animals. In line with this, a positive association between E1, the predominant estrogen type after menopause, and the loss of muscle strength has been reported [43]. Notably, age-related changes in muscle contractility were observed in female mice, with soleus muscle quality declining around the age of ovarian failure [32]. Furthermore, ovariectomy in aged female rats was shown to decrease exercise capacity and to reduce the contractile force of the soleus muscle in response to electrical stimulation [44], supporting the determinant role of estrogen loss in promoting muscle dysfunction in aged females. On the other hand, IR seems to be related to a decrease in estrogen plasma levels caused by the loss of gonadal function [45]. These results suggest that, in females, IR may impair functional decline in aged animals, possibly through alterations in the hormonal environment, highlighting the protective role of estrogens in muscle quality [42] and supporting the hypothesis of a synergistic effect among aging, metabolic dysfunction, and hormonal influences [21,23].

Moreover, neither aging nor IR significantly affected caffeine-induced muscle contraction in either the EDL or soleus across all groups evaluated. Since caffeine acts directly on ryanodine receptors in the sarcoplasmic reticulum independently of neuromuscular coupling [46], these findings suggest the absence of a defect in sarcomere-based contraction mechanisms. Therefore, the observed muscle dysfunctions are more likely attributed to impairments in neuromuscular function or upstream signaling rather than intrinsic defects in the contractile machinery itself. In line with this, age-related changes in the neuromuscular junction are increasingly recognized as a key factor in the decline of musculoskeletal function with aging [47].

Previous studies have indicated that the loss of muscle function associated with aging involves both structural and metabolic alterations in skeletal muscle [48]. Some of these alterations were further examined through histological analyses of isolated muscles.

The analysis of muscle fiber type composition is a crucial tool for understanding the functional alterations associated with both aging and IR. Succinate dehydrogenase (SDH) activity is considered a reliable marker of oxidative metabolism, allowing for the identification of distinct muscle fiber types [49]. During aging, the loss of muscle mass has been primarily attributed to the atrophy of type II fibers [50], highlighting a specific vulnerability of glycolytic fibers in this process. Furthermore, certain muscle pathologies have been shown to selectively affect specific fiber types, such as glycolytic fibers in cachexia or oxidative fibers in disuse atrophy [51,52], reinforcing the relevance of evaluating fiber distribution in different pathophysiological contexts.

In our study, SDH staining of the EDL muscles revealed a significant age-related shift in muscle fiber composition, characterized by a marked reduction in the proportion of glycolytic fibers in aged animals compared to their young controls, regardless of sex or IR status. Aged female rats exhibited this structural change despite maintaining muscle contractile function during electrical stimulation, and the presence of IR did not exacerbate the aging-related decline in glycolytic fiber percentage. These findings are consistent with previous studies reporting an age-related shift toward a more oxidative phenotype in skeletal muscle [53], likely reflecting a metabolic adaptation that enhances energy efficiency while also contributing to reduced movement speed and diminished maximal force [48].

Interestingly, a similar significant reduction in glycolytic fiber proportion was observed in young IR male and female rats compared to their respective controls. In young males, this decrease was accompanied by impaired muscle function in response to electrical stimulation, suggesting a functional consequence of fiber-type remodeling. In contrast, young IR females showed a comparable decline in glycolytic fibers without any associated functional deficits. This observation in female rats suggests that while changes in fiber type may be occurring, they might not yet be affecting muscle function, possibly due to the absence of a decline in estrogen levels. The typically higher glycolytic fiber content in males compared to females [24] may explain why their loss has a greater impact on muscle function in insulin-resistant young males.

The regenerative capacity of skeletal muscle declines with age [10,54]. Diminished regenerative capacity is recognized as one of the main contributors to muscle atrophy, sarcopenia, and functional decline associated with aging [55,56]. A histological hallmark of the failure of this regenerative attempt is the presence of myofibers with centrally located nuclei, which reflect an effort to compensate for fiber loss during aging [57,58]. In our study, we observed a significant increase in the number of muscle fibers with centrally located nuclei in both EDL and soleus muscles of aged rats, regardless of sex or IR status. This increase suggests that, during aging, skeletal muscle activates regenerative mechanisms that are incomplete or ineffective, ultimately contributing to the development of sarcopenia [10]. This feature is more closely associated with aging and does not seem to be significantly influenced by insulin resistance since young insulin-resistant animals did not show an increased number of fibers with central nuclei compared to age-matched controls across both sexes and muscle type evaluated.

Interestingly, impaired regenerative capacity has also been linked to extracellular matrix dysregulation, which further contributes to the progression of fibrosis [59]. Fibrosis is a well-established hallmark of aged skeletal muscle, characterized by the excessive accumulation of collagen and other extracellular matrix components [48,60]. Consistent with this evidence, the fibrotic area is increased in both EDL and soleus muscles of aged animals of both sexes.

Fibrosis in skeletal muscle is also closely associated with insulin resistance. Indeed, high-fat diet-induced IR has been shown to promote muscle fibrosis [61]. In our study, IR increased connective tissue content in both muscle EDL and soleus muscle from young male rats only, suggesting that metabolic dysfunction may contribute to muscle fibrosis in these animals, which also presented muscle dysfunction. The progressive replacement of contractile elements by the accumulation of fibrotic tissue could contribute to the impaired muscle strength and quality observed in aged and insulin-resistant rats [59,62,63]. The findings align with previous studies in IR mouse models, where altered responses to muscle injury and elevated collagen deposition have been reported [64]. In contrast, in young females, which displayed preserved muscle mass and function, the presence of IR did not induce a significant increase in connective tissue content, indicating a potential sex-dependent difference in the fibrotic response to IR. These observations suggest that female skeletal muscle is less susceptible to IR-induced fibrosis at early stages.

The limitations of the study include the use of a rodent model. Although widely accepted for modeling aging and insulin resistance, it presents inherent limitations when extrapolating results to human physiology. Species-specific differences may influence the applicability of our findings to clinical settings. However, among available rodent models, we selected the most appropriate one based on evidence. Extensive, side-by-side transcriptomic profiling has shown that skeletal muscle in rats is more profoundly affected by aging than in mice, and the pattern of decline observed in rats may more closely resemble that seen in humans [65]. Additionally, while muscle function was evaluated ex vivo, future functional performance evaluations are required to assess the impact of aging and insulin resistance on physical capacity.

## 4. Materials and Methods

### 4.1. Experimental Animals

Adult (3-month-old, 3 M) and aged (20-month-old, 20 M) male and female *Sprague Dawley* rats were bred in the Animal Facilities of Biomedical Research Foundation of Hospital Universitario de Getafe and housed under controlled environmental conditions (12 h light/dark cycle) with free access to tap water and a standard pellet diet. Studies were conducted following the Declaration of Helsinki and the Guide for the Care and Use of Laboratory Animals, as adopted and promulgated by the National Institutes of Health, following European regulations. All procedures were approved by the Ethics Committee for Animal Experimentation of the Hospital Universitario de Getafe (PROEX 215.5/22, approval 10 November 2022).

Fructose-fed rats were employed as a well-established and previously validated model of insulin resistance [26,27]. IR was induced by administration of D-fructose (20% *w*/*v*) dissolved in drinking water for 8 weeks, starting at 6 weeks of age for young animals and at 18 months for aged rats. Age-matched animals maintained under identical conditions but receiving normal drinking water served as controls. Experiments involving adult (3 M) and aged (20 M) rats were alternated to avoid possible sequence-dependent bias.

After treatment, rats were weighed and anesthetized with isoflurane (induction at 4%, maintenance at 2%) using an anesthesia chamber. Blood samples were collected via cardiac puncture in anti-coagulant-free tubes for serum extraction. Sera were separated by centrifugation and stored at −80 °C until biochemical measurements were made. EDL and soleus muscles from one hindlimb were extracted by dissection at the tendinous insertions for contractile measurements. In contrast, muscles from the contralateral limb were collected, flash-frozen in liquid nitrogen-cooled OCT, and stored at −80 °C for histological analyses.

The selection of the EDL and the soleus muscles was based on their distinct physiological characteristics, which allow a detailed analysis of muscle function. The soleus is a postural muscle composed mainly of slow-twitch fibers (type I), primarily involved in endurance activities and posture control. In contrast, the EDL muscle is a faster and more powerful muscle with a higher proportion of fast-twitch fibers (type II), playing a key role in rapid and high-intensity movements.

### 4.2. Biochemical Determinations

Serum concentrations of glucose, insulin (Elabscience, Houston, TX, USA), testosterone, and estradiol (Demeditec Diagnostics, Kiel, Germany) were determined using enzyme-linked immunosorbent assay (ELISA) kits, following the manufacturers’ instructions. All measurements were performed in duplicate. IR was estimated using the Homeostasis Model Assessment of Insulin Resistance (HOMA-IR) index, calculated according to the method described by Matthews et al. [66] and normalized to the mean value obtained in control animals.

### 4.3. Determination of Contractile Responses of EDL and Soleus Muscles to Electrical Stimulation

Each muscle was cut into two longitudinal strips. The contractile properties of EDL and soleus muscles were evaluated by using DMT 820 M myograph chambers (Danish Myo Technology, Hinnerup, Denmark). Muscle strips were maintained in Krebs–Henseleit (KHS) solution continuously bubbled with 95% O_2_ and 5% CO_2_ at 25 °C. The KHS had the following composition (mM): NaCl 114, KCl 4.6, CaCl_2_ 2.5, MgSO_4_ 1.2, NaHCO_3_ 24.9, glucose 11, KH_2_PO_4_ 1.2, EDTA 0.027. KHS was continuously bubbled with 95% O_2_/5% CO_2_ (pH 7.4).

Electrical stimulation was applied by means of two platinum electrodes placed at both sides of muscle strips. Parameters of electrical stimulation consisted of pulses of 1 ms duration with 100 mA of current intensity for 5 s at the indicated frequency, which were generated by an electrical stimulator (CS-420; Cibertec, Madrid, Spain) and current amplifier unit (8BO Power Unit; Cibertec). The optimal length of the muscle strips (enabling the strongest contraction in response to an electrical twitch) was established by increasing the length until the contraction response to a single electrical stimulus of 25 Hz reached its maximum. Force data were acquired, registered, and analyzed with LabChart Pro v8 software (AD Instruments, Sidney, Australia).

Once the optimal muscle length was determined, frequency–response curves were generated at stimulation frequencies of 10, 25, 50, 75, and 100 Hz, measuring the maximum contraction force for each 5 s stimulus. Additionally, contractile responses to increasing concentrations of caffeine (0.3–30 mM; Sigma Aldrich, St Louis, MO, USA) were evaluated by adding caffeine to organ chambers, and the maximal contraction of muscle strips was recorded.

### 4.4. Histological Staining

For histological analysis, 10 µm serial transverse sections were obtained from frozen EDL and soleus muscles embedded in OCT compound. Sections were cut at −20 °C by a Leica CM1860UV Cryostat (Leica Biosystems, Nussloch, Germany), mounted on polysine microscope slides (Epredia, Kalamazoo, MI, USA), and stored at −80 °C until assayed. Three different staining protocols were employed to assess specific histological features, as detailed below.

#### 4.4.1. Hematoxylin and Eosin (H&E) Staining

Tissue sections were hydrated by two consecutive 5 min washes with distilled water before H&E staining. Samples were incubated with Carazzi’s hematoxylin solution (255298.1212; AppliChem, Darmstadt, Germany) for 3 min and then rinsed twice in distilled water for 5 min. Next, sections were stained with 0.5% eosin in ethanol (251299.1608; AppliChem) for 30 s and dehydrated in increasing ethanol concentrations (80–96–100%) for 2 min each. Finally, samples were immersed in CLEAR Histo 775 (Casa Álvarez, Madrid, Spain) for 5 min and mounted using DPX mounting medium fast (Sigma Aldrich) for viewing under a microscope (Olympus BX51, Barcelona, Spain). Nuclei were stained purple, while the cytoplasmic components of muscle fibers were stained pink. At least 2 representative images per sample were captured at 100× magnification and analyzed using FIJI (ImageJ 2.6; https://fiji.sc/ (accessed on 1 July 2025)). The number of centrally located nuclei was manually counted in transverse muscle sections and expressed as the percentage of fibers containing central nuclei relative to the total number of fibers quantified per muscle.

#### 4.4.2. Succinate Dehydrogenase Assay (SDH)

The SDH activity of the fibers, which reflects their metabolism, was evaluated in frozen EDL sections. The more oxidative muscle fibers appear purple, while glycolytic fibers are lighter in color. Tissue sections were fixed in 4% paraformaldehyde for 5 min and rinsed three times in phosphate-buffered saline (PBS) for 15 min each. Then, tissue sections were incubated for 1.5 h at 38 °C in the staining solution containing (0.2 M succinate and 0.5 mM Nitro Blue tetrazolium (N5514; Sigma Aldrich) in 0.1 M phosphate buffer, pH 7.2) protected from light. Samples were washed in PBS for 15 min and finally mounted in DPX mounting medium for viewing by microscope (Olympus BX51), at least 2 representative images per sample were captured at 100× magnification and analyzed. The percentage of glycolytic fibers was quantified using FIJI (ImageJ 2.6). Glycolytic fibers, identified by their lighter SDH staining intensity, were manually counted in cross-section images and expressed as a percentage of the total number of muscle fibers analyzed per muscle.

#### 4.4.3. Masson’s Trichrome Staining

The amount of connective tissue in the EDL and soleus muscles was analyzed using Masson’s trichrome staining kit (Bio-Optica, Milano, Italy). In this staining, muscle fibers appear red, nuclei are stained dark blue or black, and connective tissue (collagen) is stained green. Tissue sections were hydrated in distilled water and stained following the manufacturer’s instructions. Sections were mounted in DPX mounting medium for viewing under a microscope (Olympus BX51). At least 4 representative images per sample were acquired at 200× magnification and analyzed using FIJI (ImageJ 2.6). The amount of connective tissue was quantified as the percentage of green-stained area relative to the total area of each muscle.

### 4.5. Statistical Analysis

Statistical analyses were performed using GraphPad Prism 8 (GraphPad Prism Software Inc., San Diego, CA, USA) and SPSS software, version 21.0 (IBM Corp., Armonk, NY, USA). Data are expressed as mean ± standard error of the mean (SEM). For comparisons among experimental groups for all variables, one-way analysis of variance (ANOVA) followed by Tukey’s test for multiple comparisons. Complete frequency–response curves from contractile studies were analyzed by two-way ANOVA followed by Bonferroni’s correction. The number of animals (n) is indicated where appropriate. Differences were considered statistically significant at *p* < 0.05.

## 5. Conclusions

In conclusion, our findings demonstrate that both aging and IR affect skeletal muscle health in a sex-dependent manner. In males, aging and IR independently impair muscle function, with aging primarily reducing contractile force and muscle mass alongside declining testosterone levels, while IR alone also compromises performance in young males. In females, aging results in muscle mass loss without a corresponding decline in function, suggesting a degree of functional preservation potentially mediated by estradiol. However, when IR co-occurs with aging, estradiol levels drop significantly, exacerbating mass loss and affecting its contractile capacity. These functional impairments are associated with underlying structural and compositional alterations, including reduced glycolytic fiber content in the EDL, impaired regenerative capacity, and increased fibrosis in both muscle types. Notably, IR reduced glycolytic fiber proportion in both sexes, but fibrosis was particularly elevated in young IR males. Together, these results highlight the complex interplay between metabolic status, sex hormones, and muscle aging, and underscore the importance of considering sex-specific mechanisms in the prevention and treatment of age- and IR-related muscle function decline. A better understanding of these interactions is essential for developing more effective and personalized therapeutic strategies aimed at preserving physical performance and health in older adults.

## Figures and Tables

**Table 1:** Biochemical and physiological parameters in male and female rats.

	3 M	3 M IR	20 M	20 M IR
**MALES**				
Body weight (g)	488.7 ± 26.06 (n = 20)	512.9 ± 30.33 (n = 15)	674.3 ± 36.02 (n = 11) ** †	661.5 ± 47.99 (n = 15) ** †
Serum FT (pg/mL)	5.419 ± 0.77 (n = 13)	6.49 ± 1.24 (n = 14)	1.64 ± 0.36 (n = 10) * ††	1.90 ± 0.48 (n = 12) * ††
% EDL mass (muscle/body weight)	0.041 ± 0.001 (n = 10)	0.042 ± 0.003 (n = 8)	0.032 ± 0.001 (n = 10) ** ††	0.030 ± 0.001 (n = 10) ** ††
% Soleus mass (muscle/body weight)	0.048 ± 0.001 (n = 11)	0.045 ± 0.002 (n = 8)	0.037 ± 0.001 (n = 8) **	0.040 ± 0.002 (n = 9) *
**FEMALES**				
Body weight (g)	286.2 ± 9.89 (n = 30)	313.1 ± 9.94(n = 22)	389.1 ± 11.74 (n = 28) *** ††	440 ± 26.37 (n = 19) *** †††
Serum estradiol (pg/mL)	27.26 ± 2.87 (n = 9)	24.23 ± 2.37 (n = 10)	21.46 ± 2.53 (n = 10)	15.68 ± 1.27 (n = 9) **
% EDL mass (muscle/body weight)	0.043 ± 0.002 (n = 10)	0.042 ± 0.001 (n = 10)	0.033 ± 0.002 (n = 9) ** ††	0.026 ± 0.002 (n = 15) *** ††† t
% Soleus mass (muscle/body weight)	0.057 ± 0.003 (n = 9)	0.055 ± 0.001 (n = 10)	0.042 ± 0.004 (n = 9) * †	0.029 ± 0.002 (n = 12) *** ††† t

Data are expressed as mean ± standard error (SEM). n indicates the number of animals per group. * *p* < 0.05, ** *p* < 0.01, *** *p* < 0.001 vs. 3 M; † *p* < 0.05, †† *p* < 0.01, ††† *p* < 0.001 vs. 3 M IR; t *p* < 0.05 vs. 20 M by one-factor ANOVA followed by Tukey’s multiple comparisons test. EDL: extensor digitorum longus; FT: free testosterone.

## Data Availability

All data are available upon request to the corresponding author.
